# Multi-UAV Routing for Area Coverage and Remote Sensing with Minimum Time

**DOI:** 10.3390/s151127783

**Published:** 2015-11-02

**Authors:** Gustavo S. C. Avellar, Guilherme A. S. Pereira, Luciano C. A. Pimenta, Paulo Iscold

**Affiliations:** Escola de Engenharia, Universidade Federal de Minas Gerais, Av. Antônio Carlos 6627, Belo Horizonte 31270-901, MG, Brazil; E-Mails: gustavoavellar@ufmg.br (G.S.C.A.); lucpim@cpdee.ufmg.br (L.C.A.P.); iscold309@gmail.com (P.I.)

**Keywords:** coverage path planning, UAVs, vehicle routing problem

## Abstract

This paper presents a solution for the problem of minimum time coverage of ground areas using a group of unmanned air vehicles (UAVs) equipped with image sensors. The solution is divided into two parts: (i) the task modeling as a graph whose vertices are geographic coordinates determined in such a way that a single UAV would cover the area in minimum time; and (ii) the solution of a mixed integer linear programming problem, formulated according to the graph variables defined in the first part, to route the team of UAVs over the area. The main contribution of the proposed methodology, when compared with the traditional vehicle routing problem’s (VRP) solutions, is the fact that our method solves some practical problems only encountered during the execution of the task with actual UAVs. In this line, one of the main contributions of the paper is that the number of UAVs used to cover the area is automatically selected by solving the optimization problem. The number of UAVs is influenced by the vehicles’ maximum flight time and by the setup time, which is the time needed to prepare and launch a UAV. To illustrate the methodology, the paper presents experimental results obtained with two hand-launched, fixed-wing UAVs.

## 1. Introduction

The world is on the verge of a major breakthrough, as we reach a moment in history when UAV flights become regulated in many countries around the world. Companies from different fields are currently using UAV and sensor technologies to acquire information of ground regions and to reduce the time and costs of operation. Applications, such as environment monitoring, search and rescue, precision agriculture and surveillance, may benefit from this usage of UAVs with onboard sensors for spatial coverage [1,2,3,4].

This work, which was motivated and mainly financed by FINEP (Funding Agency for Studies and Projects), a funding agency of the Brazilian government, deals with one of the most common uses of aerial robot technologies, which is the one for obtaining a series of overlapping aerial images from the ground. These images are usually post-processed for the extraction of desired information, such as digital terrain maps and vegetation indexes. In this context, efficient UAV path planning algorithms are of great importance, since the operation time, costs and the quality of the information extracted from the images are directly related to the quality of such a planning. We propose an area coverage path planning strategy to obtain images of the ground considering a multi-UAV scenario.

Several area coverage strategies have been proposed in the literature. A comprehensive recent survey of methods can be found in [5]. The large majority of strategies rely on decomposing the target area into cells that must be visited and covered. Choset [6], for example, proposed a method of exact cellular decomposition dedicated to coverage tasks. The method divides the space in convex regions that must be covered by a sequence of back and forth movements. The cells are modeled as nodes in an adjacency graph in which edges represent the existence of a common boundary between two cells. The coverage problem is then solved by first executing a graph search to determine in which order the cells should be covered. Second, the robot moves from cell to cell according to the specified sequence, performing the back and forth movements inside each cell to cover it entirely. Acar *et al.* [7] adapted this method to work on smooth and polygonal workspaces. Instead of searching for vertices, the proposed algorithm looks for connectivity changes in the workspace. Acar *et al.* [8] uses this method and improves it by describing each cell as narrow or vast. In vast cells, the back and forth movements are executed as usual, with the width of each coverage row proportional to the size of the footprint associated with the sensor carried by the robot. On the other hand, in narrow cells, the robot changes its behavior and uses its sensor to follow the associated generalized Voronoi diagram (GVD) to cover the cell.

The only optimization involved in the previously mentioned methods is in the choice of the order of cells to be followed. A simple form of path optimization is to choose the direction of the back and forth movements in each cell. This may reduce the number of turns in the path, thus reducing the effects of vehicle deceleration and acceleration due to each turn [9]. Li *et al.* [10] takes this method one step further by also addressing the connection between cells. In [10], the authors were able to prove that, for UAVs, a path with less turns is more efficient in terms of route length, duration and energy. The optimization of the sweep direction was also applied in the context of multi-UAV systems in [11]. In this case, the target area was partitioned into convex polygons, and each one of these polygons was assigned to a UAV, which had the responsibility of covering the area by following a back and forth pattern according to the optimal sweep direction.

Xu *et al.* [12] presented an area coverage strategy that combines the ideas of the exact cell decomposition of Choset [6] with the direction of coverage optimization suitable for single fixed-wing UAV operation. As the authors use a graph representation (Reeb graph), where the cells are not modeled as nodes, but as graph edges, the sequence of cells is found by solving the so-called Chinese postman problem (CPP), which is a routing problem in which the objective is to find the shortest tour that visits every edge at least once.

Some researchers have focused on solutions that take into account multiple robots, such as the work in [11] that was previously mentioned. The use of multi-robot systems has several advantages, such as the reduction of mission time due to workload division and the introduction of fault tolerance, as one robot may cover the region initially assigned to another robot in case of failure. In this context, a common approach is to consider the coverage problem as a vehicle routing problem (VRP).

In general, a VRP is the problem of finding a set of routes to be executed by a set of vehicles that must visit a set of customers at different geographical locations, given a transportation road network. These routes must fulfill all of the customers’ demands of goods, satisfy the operational constraints and minimize an objective function that reflects the global transportation cost [13]. The transformation of the coverage problem into a VRP is usually carried out by building a graph in such a way that coverage is reached when a set of nodes or edges of this graph is visited at least once by a robot. The different solutions in this line vary in at least one of the following aspects: the form of constructing this graph, the manner of obtaining coverage, *i.e.*, which nodes or which edges must be visited, the operational constraints imposed on the robots and the objective function to be minimized.

In [14], the authors consider a multi-UAV routing problem with the possibility of modeling periods of loitering and also dealing with general relative timing constraints. In their formulation, it is possible to model scenarios in which a waypoint has to be visited more than once with given timing constraints. The problem of boundary coverage by a multi-robot system is addressed in [15]. In this case, the objective is to generate balanced inspection routes that cover a subset of the graph edges instead of the graph nodes. The work in [16] solves the problem of planning routes to multiple UAVs with the collected amount of information from desired regions being the objective function to be maximized. The information is acquired by down-facing cameras installed on the UAVs, and the computation of the information takes into account the variation of the resolution at different parts of the captured image. Dynamic vehicle routing with the objective of spatial and temporal coverage of points of interest, modeled as nodes in a graph, is the focus of [17]. The spatial coverage is related to the fact that the points are distributed over the area, and the temporal coverage is associated with the existence of time constraints determining when the points have to be covered. The environment is dynamic in the sense that the targets evolve spatially and temporally. Three conflicting objectives are considered simultaneously: (i) minimization of the distance traveled; (ii) maximization of satisfaction, which models the necessity of covering the targets within given time windows; and (iii) minimization of the number of UAVs. A vehicle routing problem with multiple depots is the model used in [18]. In the VRP nomenclature, the depot is the place where the vehicles start and finish their tasks. The objective in [18] is the minimization of the longest tour performed by every UAV, which is equivalent to the minimization of the total mission time.

This paper presents a methodology for optimal time coverage of ground areas using multiple fixed-wing UAVs. Similar to other works, we solve the coverage problem by creating a graph and transforming the original problem into a vehicle routing problem. The main contribution of this work is the incorporation of specific features that are relevant in a real-world deployment. We assume the common scenario in which the number of human operators responsible for launching and retrieving the UAVs is smaller than the number of vehicles. This is incorporated in the method by defining a so-called setup time. In some situations, the setup time prevents two UAVs from being launched within small intervals of time. This means that, since one operator cannot prepare more than one UAV at the same time, the setup time of each UAV is cumulative. For example, consider a mission with one operator, two UAVs and a setup time of 4min. The pre-flight tasks for the first UAV will take 4min and will take 8min for the second UAV, 4min of which the operator was working on the first UAV while the second was idle. Similar to [18], in this work, we aim to solve the multi-UAV coverage task in minimum time, and in the same spirit of [17], it is also our goal to use a reduced number of vehicles, if possible. Our method finds the optimal routes and number of necessary UAVs automatically considering the number of human operators available. Given the constraint on the number of operators, there are scenarios in which launching a large number of UAVs may have a negative impact in the total mission time, due to the influence of the cumulative setup time. Furthermore, in order to reduce the number of turns during the mission, we also optimize the sweep direction as in [9]. We evaluate the method in a real-world experiment with two actual aerial vehicles.

This paper is organized as follows. Section 2 presents the problem statement. The proposed solution is presented in Section 3. Experimental results in simulated and real environments using multiple UAVs are shown in Section 4 and Section 5, respectively. The conclusions and some perspectives for future works are presented in Section 6.

## 2. Problem Definition

In this paper, it is assumed that a group of *M* fixed-wing UAVs has the mission to cover, as quickly as possible, a polygonal convex area represented by a set *P* of vertices in $R^{2}$. If a non-convex area is to be covered, it is assumed that *P* represents the convex hull of the area. It is considered that the maximum time of flight of each UAV is finite and known in advance. Each UAV is equipped with an on-board camera pointing down. The mission of the UAVs is to sense, using the on-board cameras, the entire region specified by *P*. The altitude of the UAVs flight is constant and is carefully chosen so that the resolution of the camera allows the observation of the characteristics of interest on the ground. We assume that all UAVs are identical in terms of hardware and power, although this is not a limitation to our methodology. Related to the operation of the UAVs, we assume the necessity of a setup time before the flight of each UAV. This setup time includes the connection of the batteries, the GPS fixing and the launching itself, among other tasks.

Given this, the specific problems we are dealing with in this paper are: (i) to discover the number m≤M of UAVs that minimize the time taken to cover the area represented by *P*; and (ii) to specify the paths for each UAV so that the mission is completed in minimum time. Notice that, given the setup time, the ideal number of UAVs cannot be trivially chosen to be *M*. In the same way, the setup time and the dynamic constraints of the UAV also prevent the area from being simply divided among the available UAVs. The next section will present our solution to these problems.

## 3. Methodology

Our strategy for solving the problem presented in the previous section is divided into two parts. In the first part, we decompose the area to be covered as a set of sweeping rows, using a methodology similar to the one proposed in [9]. These rows form the edges of a graph that is used in the second part of the method, which is based on a vehicle routing problem solution. The next subsections detail each part of the method.

### 3.1. Area Decomposition

**Figure 1:**
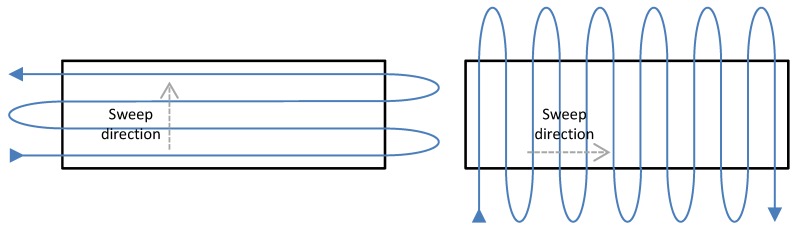
Coverage strategy used in this work. A rectangular area is covered using a back and forth motion along lines perpendicular to the sweep direction. Notice that the sweep direction highly influences the number of turns outside the area to be covered, thus affecting the coverage time. The optimal sweep direction is parallel to the smallest linear dimension of the area [9].

In this work, we assume that the UAVs will fly over the area to be covered executing a back and forth motion in rows perpendicular to a given sweep direction, as shown in Figure 1. While following the rows, the UAV is leveled (which means that its camera is pointing down), but at the end of the row, it makes a curve outside the area to return to the next row. During such a curve, the camera is generally not pointing to the ground. Furthermore, as pointed out by Huang [9], the number of turns is directly related to the time of coverage of a given region. Therefore, as suggested in [9], the first step of our method is to find the optimal direction of coverage, which is perpendicular to the smallest height of the polygonal area. In this direction, the area can be covered with the smallest number of rows, thus with the smallest number of curves. This can be observed in Figure 1. As pointed out by [10], a path with less turns is more efficient in terms of route length, duration and energy. However, notice that the sweep direction may be chosen in different ways. It is possible, for example, to choose the direction of the coverage rows as a function of the wind, since flying against the wind may destabilize the vehicle [12].

**Figure 2:**
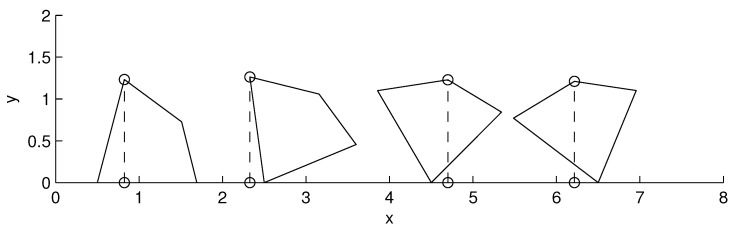
Procedure to search the optimal direction of coverage. The area is rotated until the smallest height is found.

To find the optimal direction of coverage of a given polygon, a simple search procedure may be used. As shown in Figure 2, the polygon is rotated over a surface, and its height is measured. The best orientation is the one that yields the smallest height, $h_{min}$.

**Figure 3:**
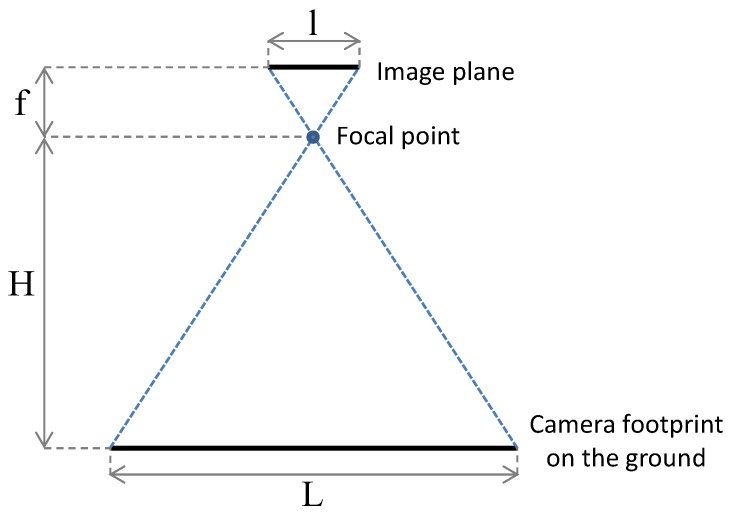
Relation between the size of the camera sensor, the height of flight and the camera footprint on the ground.

Once the optimal sweep direction is found, it is then possible to distribute the rows over the area. In our approach, the distance between two rows is chosen as a function of the footprint of the on-board cameras on the ground. As shown in Figure 3, assuming that the image sensor is parallel to the ground plane (*i.e.*, the UAV is leveled), by knowing the width of the image sensor, *l*, the focal distance of the camera’s lens, *f*, both in millimeters, and the distance between the camera and the ground, *H* (the flying height), in meters, it is possible to compute the width *L* of the camera’s footprint, in meters, as:

(1) $$L=H\frac{l}{f}\;$$

The number of coverage rows is then computed as:

(2) $$N_{l}=\left\lceil \frac{h_{min}}{L(1-s)}\right\rceil \;$$

while the distance, in meters, between two rows is:

(3) $$d_{l}=\frac{h_{min}}{N_{l}}$$

where s∈(0,1) represents the fraction of overlap between two images. This overlap is generally necessary to concatenate the images to compose an aerial map.

Assuming that the polygon that represents the region is rotated in a way that the optimal direction of coverage is parallel to the *x* axis of the global reference frame, the coverage rows can be defined by two planar points (x,y) with identical *y* coordinates given by:

(4) $$y_{i}=i\times d_{l}-\frac{d_{l}}{2},\;i=1,\ldots ,N_{l}\;$$

and *x* coordinates defined by the points where the horizontal straight line with coordinate $y_{i}$ intercepts the borders of the area to be covered. Once the points are computed, they are rotated back to the original orientation.

**Figure 4:**
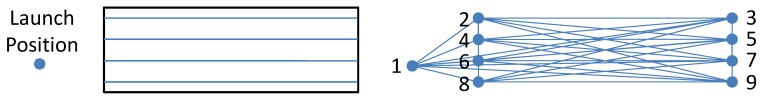
Graph representing the coverage problem. On the left, a rectangular region to be covered, the launch position and the covered rows. The nodes of the graph on the right are composed of the launch position and the intersection points between the coverage rows and the borders of the region. All nodes are connected by edges, forming a complete graph.

The extreme points of the coverage rows, along with the coordinates of the UAV launch position, called the base or depot, are considered to be the set of nodes *V* of a graph G=(V,E). Each node of the graph is numbered so that the base receives Number 1, the nodes related to the first coverage row receives Numbers 2 and 3, the ones associated with the second row are labeled 4 and 5, and so on. At the end, each coverage row is associated with subsequent even and odd nodes. The edge set, *E*, is composed of all lines connecting the *N* nodes of the graph, thus forming a complete graph, as shown in Figure 4.

Mathematically, graph *G* may be represented by an N×N cost matrix *C* whose elements, $C_{ij}$, are given by the Euclidean distance between the spatial coordinates of nodes *i* and *j*. It is important to notice that *C* is time invariant, symmetric, *i.e.*, $C_{ij}=C_{ji},\forall \left(i,j\right)\in E$, and its elements satisfy the triangular inequality, *i.e.*, $C_{ij}+C_{jk}\geq C_{ik},\forall \left(i,j\right),\left(j,k\right),\left(i,k\right)\in E$. The next subsection will describe how the graph represented by *C* will be used in an optimization problem to allow the coverage of an area in minimum time using multiple UAVs.

### 3.2. Routing Strategy

Once a graph associated with the region to be covered is created, the coverage problem can be posed as a vehicle routing problem (VRP) [13]. In this class of problems, a set of customers must be visited by a set of vehicles. To transform the problem proposed in Section 2 into a VRP, each UAV will be modeled as a vehicle and each extreme point of the coverage rows as a customer. Furthermore, by proposing new constraints, it is possible to enforce the vehicles to use some pre-specified edges of the graph in their routes, so that the coverage rows will be certainly followed by one of the launched UAVs. Finally, by solving the VRP, one can obtain the set of routes that each UAV will have to perform.

Before presenting the mathematical formulation of the routing problem, we will define the constants and variables necessary for this formulation. As defined in the previous section, constant $C_{ij}$ represents the traversing cost of the edge (i,j) between nodes *i* and *j*. To indicate whether or not the k-th UAV is going to fly from vertex *i* to vertex *j*, the binary variable $X_{ij}^{k}\in \left\{0,1\right\}$ is used. Furthermore, let constant $V_{ij}^{k}\in R$ represent the flight speed of the k-th UAV while flying from vertex *i* to vertex *j*, constant $t_{s}\in R$ be the individual setup time and $L_{k}$ be the battery duration of UAV number *k*. Let also m∈N be the variable that represents the number of UAVs designed for a mission, M∈N be the total number of UAVs available, O∈N be the constant number of UAV operators and N∈N be the number of nodes of the graph. Finally, the variable $d_{k}$ represents the extra time necessary to launch UAV *k*.

Based on the variables previously defined, the time spent by UAV *k* to fly its route is mathematically given by:

$$T_{k}=\sum_{i=1}^{N}\sum_{j=1}^{N}\frac{C_{ij}}{V_{ij}^{k}}X_{ij}^{k}+d_{k}\;$$

Our main objective is to minimize the mission time. This can be accomplished by minimizing the time of the longest route among the routes of all UAVs. Therefore, our problem is in fact a min-max problem in which we want to minimize the maximum $T_{k}$. To transform the min-max problem into a linear problem, we introduce an extra variable V, which represents the longest UAV route. The basic optimization problem is then written as:

(5) min(V)

subject to

(6) $$\sum_{i=1}^{N}\sum_{j=1}^{N}\frac{C_{ij}}{V_{ij}^{k}}X_{ij}^{k}+d_{k}\leq V,\;k=1,\ldots ,M$$

(7) $$t_{s}\left\lceil \frac{k}{O}\right\rceil \sum_{j=1}^{N}X_{1j}^{k}=d_{k},\;k=1,\ldots ,M$$

As previously shown, constraint in Equation (6) accounts for the individual cost (time) of UAV *k*. This corresponds to $T_{k}$. By defining this constraint along with the objective function in Equation (5) using variable V, we are posing a linear version of the min-max problem, where the maximum cost among the ones of all UAVs must be minimum. By doing this, we are, in practice, minimizing the time taken to cover the complete area.

In Equation (6), the term $d_{k}$, detailed in Equation (7), accounts for the setup time, $t_{s}$, which is an extra cost that corresponds to the time spent by a human operator to prepare and launch the UAV for the mission. In a mission where only one person is operating the whole team of UAVs, each UAV will have the extra time $d_{k}$, which will be cumulative. In a system with two UAVs, for example, while UAV 1 is prepared, nothing is happening with UAV 2. After the launch of UAV 1, UAV 2 is prepared to be launched. Notice in Equation (7) that $d_{k}$ is null when the UAV is not used, once $X_{1j}^{k}$ is zero, indicating that UAV *k* did not leave Node 1, which represents the launching position.

Since the setup time is one of the main contributions of this work, before proceeding with the additional constraints necessary to guarantee a coverage solution, we will explore the effect of constraint in Equation (7) with three examples. For the first example, assume a team of M=3 UAVs with individual setup time $t_{s}=10\;$min and a single operator (O=1). The team is supposed to cover a rectangular area with 8 coverage rows. Each row can be covered in 2.5min, and the time to reach the region and to change between rows is considered to be negligible. Given this, the cumulative setup time of each UAV, as computed by Equation (7), is given by:

$$\begin{array}{cc} t_{s}\left\lceil \frac{k}{O}\right\rceil \sum_{j=1}^{N}X_{1j}^{k} & =d_{k},\;k=1,\ldots ,M\;\\ 10\times \left\lceil 1/1\right\rceil \times 1 & =10=d_{1}\;\\ 10\times \left\lceil 2/1\right\rceil \times 1 & =20=d_{2}\;\\ 10\times \left\lceil 3/1\right\rceil \times 1 & =30=d_{3}\; \end{array}$$

Notice that the cumulative setup time for UAV 3, $d_{3}=30\;$min, is equal to the time a single UAV would expend to cover the region (8×2.5+10min). This indicates that the use of 3 UAVs for this mission is not worth it. In this way, the best solution would make $X_{1j}^{3}=0$, indicating that UAV 3 would not be used in this mission. Two UAVs would be launched, and the best coverage time would be 25min. For this solution, UAV 1 would cover six coverage rows and UAV 2 only two rows. As a remark, if three UAVs were considered, the best coverage time would be 32.5min.

The second example explores the case when the number of operators is larger than one, but is smaller than the number of UAVs available. Suppose a team of M=5 UAVs with individual setup time $t_{s}=10\;$min and a number of O=2operators. Using Equation (7), we have:

$$\begin{array}{cc} 10\times \left\lceil 1/2\right\rceil \times 1 & =10=d_{1}\;\\ 10\times \left\lceil 2/2\right\rceil \times 1 & =10=d_{2}\;\\ 10\times \left\lceil 3/2\right\rceil \times 1 & =20=d_{3}\;\\ 10\times \left\lceil 4/2\right\rceil \times 1 & =20=d_{4}\;\\ 10\times \left\lceil 5/2\right\rceil \times 1 & =30=d_{5}\; \end{array}$$

As can be seen, UAVs 1 and 2 have the same setup time, because the two operators will prepare them simultaneously. After the takeoff of these UAVs, UAVs 3 and 4 will be prepared. Their setup time is 20min, composed of 10min of waiting for UAV 1 and 2 to be prepared and 10min of their own preparation. For UAV 5, the same reasoning can be applied.

In a third example, consider the case where the number of operators is equal to the number of UAVs. In this case, since k/O in Equation (7) will be equal to one for all UAVs, the setup time for each UAV would be $t_{s}$.

To complete the optimization problem and to guarantee its solution, a solution to the problem posed in Section 2 is indeed, and other constraints need to be incorporated into the the basic problem. The first of these constraints is given by:

(8) $$\sum_{i=1}^{N}\sum_{j=1}^{N}\frac{C_{ij}}{V_{ij}^{k}}X_{ij}^{k}\leq L_{k},\;k=1,\ldots ,M\;$$

which limits the maximum time of flight of UAV *k* by its battery duration, $L_{k}$. We consider that the charge of the battery does not decrease during the setup time or that charge decreasing is negligible. In this way, the time of flight of each UAV is simply the total time in Equation (6) subtracted by the setup time $d_{k}$. It is important to mention that constraint in Equation (8) can make the problem infeasible. This is expected if, for example, a large area is to be covered by a very small team of UAVs. The only solution in this case would be to increase the number of agents. In another situation, if the time to cover a single row is larger than the battery duration of a single UAV, the problem would also have no solution. In this case, one could go back to the first step of the methodology and try to reduce the length of the rows by changing the sweep direction (which would increase the number of turns). A more complex alternative for both situations would be to change the optimization problem to consider battery recharging. These solutions are left as suggestions for future research.

To guarantee that each node of the graph is visited only once by a single UAV, two other constraints are necessary:

(9) $$\begin{array}{cc} \sum_{k=1}^{M}\sum_{i=1}^{N}X_{ij}^{k} & =1,\;j=2,3,4\ldots ,N\; \end{array}$$

(10) $$\begin{array}{cc} \sum_{i=1}^{N}X_{ip}^{k}-\sum_{j=1}^{N}X_{pj}^{k} & =0,\;p=1,2,3\ldots ,N,\;k=1,2,3\ldots ,M\; \end{array}$$

Notice that constraint in Equation (9) enforces that each node, except the base (represented by Node 1) is visited by only one UAV. On the other hand, constraint in Equation (10) guarantees that the UAV that arrives at a given node is the same one that leaves this node.

To enforce that each UAV path starts and finishes at the base (Node 1) and to guarantee that the path has no internal cycles, a standard sub-tour elimination constraint [19] is used:

(11) $$u_{i}-u_{j}+N\sum_{k=1}^{M}X_{ij}^{k}\leq N-1,\;i,j=2,3,4\ldots ,N$$

where $u_{i}\in Z,i=2,3,4,\ldots ,N$.

To make sure the VRP solution will make the UAVs cover the area modeled by graph *G*, the following constraint is also necessary:

(12) $$\sum_{k=1}^{M}X_{i,i+1}^{k}+\sum_{k=1}^{M}X_{i+1,i}^{k}=1,\;i=2,4,6\ldots ,N\;$$

This constraint enforces that each UAV, having visited one of the nodes of a coverage row, must visit the other node of that row. This is possible given the way the nodes were numbered (see Figure 4). Constraint in Equation (12) guarantees that each UAV that visits an even node also visits the next odd node. Furthermore, a UAV that visits an odd node must visit the previous even node. Therefore, this constraint is essential to make the problem solution an actual coverage solution.

To avoid the UAVs crossing the coverage area following an edge that is not parallel to the coverage rows, two optional constraints can be added to the optimization problem:

(13) $$\begin{array}{cc} \sum_{k=1}^{M}X_{i,i+1}^{k} & =\sum_{k=1}^{M}\sum_{j=\{1,3,\ldots \}\backslash \{i+1\}}^{N}X_{i+1,j}^{k},\;i=2,4,6\ldots ,N \end{array}$$

(14) $$\begin{array}{cc} \sum_{k=1}^{M}X_{i,i-1}^{k} & =\sum_{k=1}^{M}\sum_{j=\{1\}\cup \{2,4,\ldots \}\backslash \{i-1\}}^{N}X_{i-1,j}^{k},\;i=3,5,7\ldots \;.N\; \end{array}$$

In practice, constraints in Equations (13) and (14) can avoid the UAVs executing sharp turns and also photos being taken in different directions. If these issues are not important for a given task, these two constraints can be simply ignored without compromising the execution of the task.

Finally, to allow the number of UAVs used in the mission, *m*, to be smaller than the maximum number of UAVs available, *M*, we introduce the following constraints:

(15) $$\sum_{k=1}^{M}\sum_{j=1}^{N}X_{1j}^{k}=m$$

(16) m≤M

It is important to mention that constraints in Equations (15) and (16) represent an important contribution of this work in relation to others that were previously published, such as [19], where the number of UAVs is kept constant. In this work, the number of UAVs will be chosen as a function of the minimization of V.

By solving the problem represented by the objective function in Equation (5) and constraints in Equations (6)–(16), one would expect to obtain a solution in which the minimum number of UAVs would follow the shortest possible paths, covering in minimum time the area modeled by graph *G*. However, although the mission time will indeed be minimum, the objective function does not explicitly take into account the number of UAVs, which may cause the computed solution to consider a number of UAVs that is not optimal. Moreover, since our problem minimizes the longest path in terms of mission time, once the best longest path is found, there is no guarantee that the paths for the other UAVs are minimized. To make sure that the optimal number of UAVs, *m*, is chosen and that the paths for all UAVs are minimum, two strategies were devised.

The first one is a heuristic and depends on the adjustment of some constants. The idea is to change the utility cost function, so that it explicitly takes into account the number of UAVs and/or a combination of all UAV path costs. For example, the cost function V+ρm explicitly considers the number of UAVs. A correct choice of constant ρ will make the optimizer find the best *m*. In the same way, if $T_{k}$ is the individual coverage time for UAV *k*, the cost function $V+\rho \;mean(T_{k})$ would minimize the paths of all UAVs if ρ is chosen properly.

The second strategy, which will generate the optimal solution in terms of the number of UAVs and individual paths independently of parameter choices, is an iterative solution that consists of solving the optimization problem more than once. In the first iteration, the original graph is used, and the optimization problem to be solved is exactly the one previously presented. In the second iteration, the problem is reduced by removing the UAV assigned to the longest path in the first iteration, which is already optimal, and all nodes (except for the base) and associated edges that belong to its path. This procedure is repeated until all nodes, but the base, are removed from the graph. With this procedure, we have a better use of the available resources. It is then guaranteed that the optimal number of UAVs is found and that the paths for all UAVs are optimal without compromising the primary objective, which is to minimize the cost of the route with the highest cost.

In the next section, we present simulations that illustrate our methodology and the role of the constraints in the optimization problem.

## 4. Simulations

This section intends to illustrate the proposed methodology using a series of simulations. All simulations were executed in MATLAB on an Intel Core i5 1.7 GHz computer with 4 GB of RAM. The optimization problem was solved using the Yalmip [20] toolbox in the front-end and the Gorubi [21] solver in the back-end.

**Figure 5:**
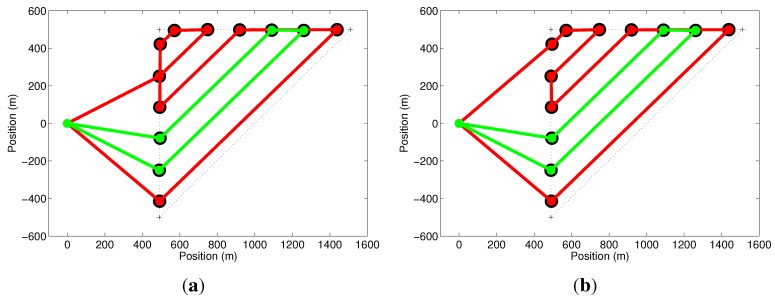
Effect of the cost function. (**a**) The mission represented by the green path is optimized, but the other (red) is not the minimum; (**b**) By removing the largest path and running the optimizer again, all paths are optimized.

In our first simulation, we explore the cost function in Equation (5) and some of the issues mentioned before, related to the optimization of the paths of all UAVs. This simulation consists of two UAVs with a setup time of 2min being deployed on a mission to cover a triangular-shaped area. Figure 5a shows that the green path, which corresponds to a mission of 8.16min, was optimized in the first iteration. The red path, corresponding to a mission of 8.13min, has unnecessary cycles and is not the minimum. This problem is solved by running the optimization algorithm once again with the removal of the first UAV and its path, as shown in Figure 5b. In this case, the UAV with the red path takes the reduced time of 8.03min to finish its mission.

**Figure 6:**
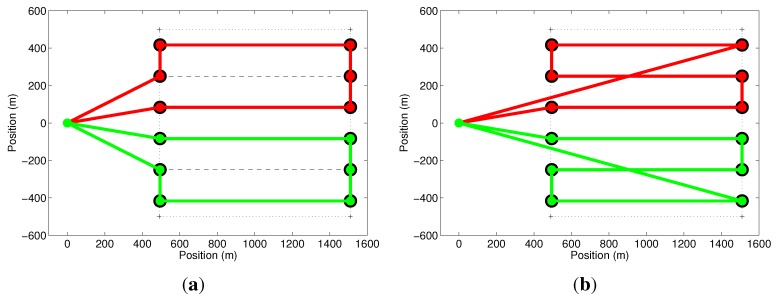
Effect of constraint in Equation (12). The solid lines represent the paths for the two UAVs used in the simulation. Optimization results without (**a**) and with (**b**) the constraint.

Our second set of simulations explores the activation of constraint in Equation (12). To show the effect of this constraint, we have used two UAVs to cover a square-shaped area with no setup time, as shown in Figure 6. Figure 6a shows, in the solid line, the paths of the UAVs when constraint in Equation (12) is removed. The paths when this constraint is considered are shown in Figure 6b. Notice that the result shown in Figure 6a is a solution for a multiple traveling salesman problem (mTSP), but is not an area coverage solution.

**Figure 7:**
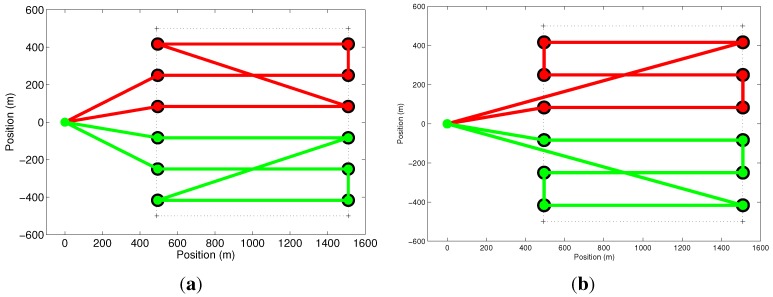
Effect of constraints in Equations (13) and (14). The solid lines represent the paths for the two UAVs used in the simulation. Optimization results without (**a**) and with (**b**) the constraints.

The next simulation shows the effect of constraints in Equations (13) and (14). In the result shown in Figure 7a, these constraints were removed. If we compare this result with Figure 7b, where the constraints are in place, it is possible to see that the constraints do not allow the UAV to move from one node to another, crossing the coverage region, except when the UAV is moving from or to the base. It is important to mention that the path obtained without the constraints takes 7.11min to be followed by each UAV, while the path found with the constraints is longer and can be followed in 7.26min. On the other hand, the problem can be computed in 4.59s without the constraints and in 0.8s with the constraints. This happens because these constraints act as if they are removing the diagonal edges of the graph, which, in fact, reduces the size of the problem. Thus, observe that the use of constraints in Equations (13) and (14) can be avoided if the diagonal edges are removed from the original graph.

**Figure 8:**
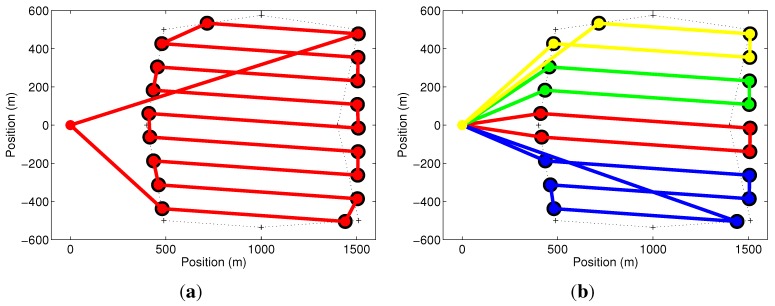
Two trivial solutions for the optimization problem. (**a**) The path for the only UAV available; (**b**) the paths for the four UAVs available when the setup time is not considered or each UAV has it is own operator (the setup time is noncumulative). In those cases, the area is simply divided among the available vehicles.

In what follows, we show the effects of constraint in Equation (7). For this set of simulations, a non-convex area is selected, and the methodology is applied to the area’s convex hull. Results shown in Figure 8 represent trivial solutions for this area, when the setup time is not important. Figure 8a shows the optimization results for a mission when only one UAV was available (M=1). In this case, the UAV took 20.8min to complete the coverage, including 4min of setup time. Figure 8b shows the optimization results for a mission when four UAVs were available (M=4), each UAV with its own operator. This means that there is no cumulative setup time, and each UAV has only the original 4min of setup time. Notice that the optimizer tries to equally divide the coverage area among the UAVs. In this simulation, the four UAVs took, respectively, 8.21, 8.36, 11.10 and 8.64min to perform their tasks, including the setup time. A similar result would be found if the setup time were set to be null.

**Figure 9:**
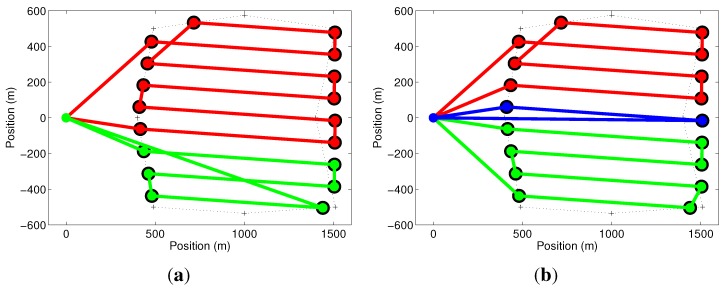
(**a**) Optimal solution for the problem in Figure 8 when the four UAVs available are operated by a single person and the cumulative setup time is considered. Only two UAVs were selected by the method; (**b**) Effect of constraint in Equation (8) when the UAVs’ battery life is reduced and a third UAV is necessary to complete the task.

For the same coverage area and number of UAVs (M=4), but only one human operator, the cumulative setup time in Equation (7) must be considered. Figure 9a shows that the proposed methodology, in this case, found that the problem is best solved with two UAVs (m=2). In this result, the UAVs take 14.73 and 15.10min to complete their missions, including 4 and 8min of setup time, which is the optimal solution in this case. To show that this result is correct, we force the use of a third UAV by exploring the effect of constraint in Equation (8), which is related to the duration of the batteries. We have reduced the UAVs’ maximum flight time from to 30 down to 10min. This means that, if the previous solution is considered, none of the UAVs would be able to complete their missions. Using Equation (8), the optimizer automatically selects a third UAV, making m=3, to ensure that the mission will be completed. This solution is shown in Figure 9b, where the mission times for each UAV are 11.54, 15.51 and 16.03min, including 4, 8 and 12min of setup time. Notice that this result is worse than the one with two UAVs, which cannot be used in practice due to the reduced flight time. Furthermore, observe that the times of flight of each UAV, computed as the mission times minus the setup times, are always smaller than the battery life (10min).

**Figure 10:**
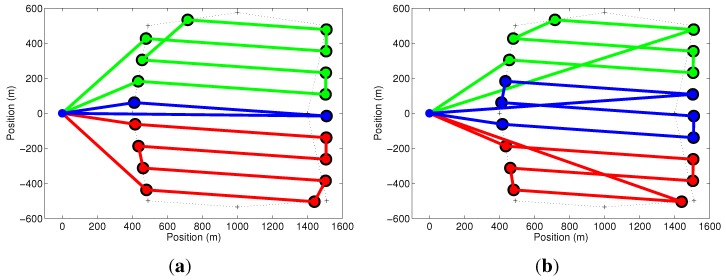
(**a**) Optimal solution for the problem in Figure 8 when the four UAVs available are operated by a team of two operators. Only three UAVs were selected by the method; (**b**) Optimal solution for the problem in Figure 8b when the four UAVs available are operated by a team of three operators. Again, only three UAVs were selected by the method.

For the last set of simulations, we explore the effect of a number of operators larger than one and smaller than the number of available UAVs, using the same coverage area and number of UAVs (M=4) of the previous simulations, but returning the UAVs’ maximum flight time to 30min. Two simulations were made, with teams of two and three operators. In both cases, the number of UAVs selected by the method was m=3. Figure 10a shows that the proposed methodology found a result similar to the one in Figure 9b when two operators were available. In this result, the UAVs take 11.51, 11.54 and 12.03min to complete their missions, including 4min of setup time for the first two UAVs and 8min of setup time for the third one. Despite having the same flight path, mission times are smaller than those in Figure 9b, because the first two UAVs are simultaneously prepared and launched. The third UAV became more productive, having more flight time, when a third operator was included for the team, as shown in Figure 10b, where the mission times for each UAV are 11.10, 11.19, and 11.26min, including 4min of setup time for each one of them. Notice that this result is one of the best shown in this paper for this specific area. In the best result, shown in Figure 8b, the longest UAV route took 11.10min, but this solution employed a fourth UAV and fourth operator, which probably would not be worth a gain of 0.16min or 9.60s.

## 5. Real-World Experiments

**Figure 11:**
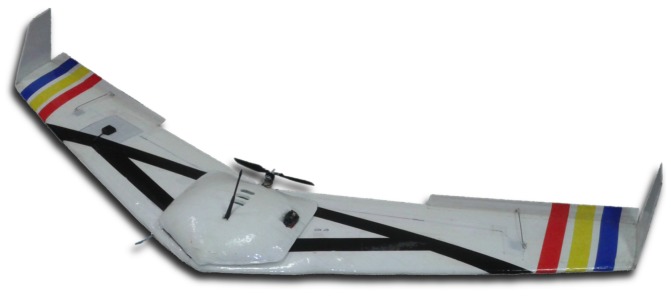
Picture of one of the UAVs used in the experiments.

**Figure 12:**
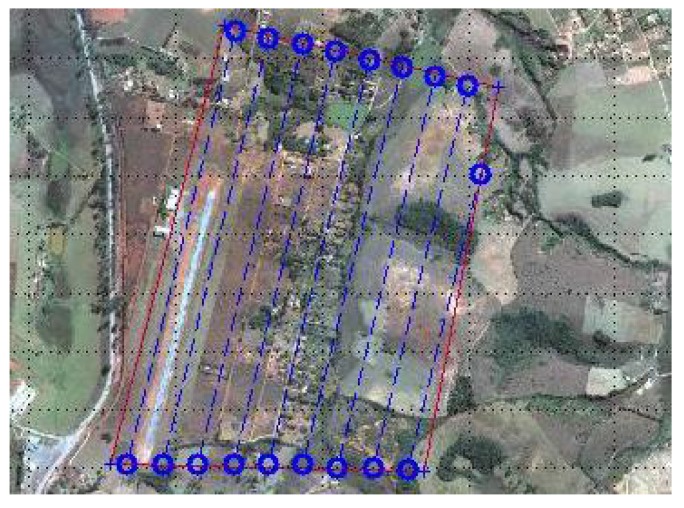
Coverage area used in the experiments overlaid on a satellite image.

We have tested our methodology in practice using two fixed-wing UAVs controlled by the 2128${}^{\text{g}}$ Micropilot’s autopilot. A picture of one of these UAVs is shown in Figure 11. Each UAV was equipped with a Canon Powershot ELPH130 camera with sensor of 6.17 mm in width and focal length of 5.0 mm. The camera was pointing down. This testbed is described in detail in [22]. Considering that the task would be executed at a height of 120m and that an overlap of 30% between two consecutive images is required, we use Equation (1) to find the distance between coverage rows to be 105m. The area to be covered was chosen to have approximate dimensions of 900m×1600m, as shown in Figure 12, where the area and the coverage rows are overlaid on a Google Earth satellite image.

**Figure 13:**
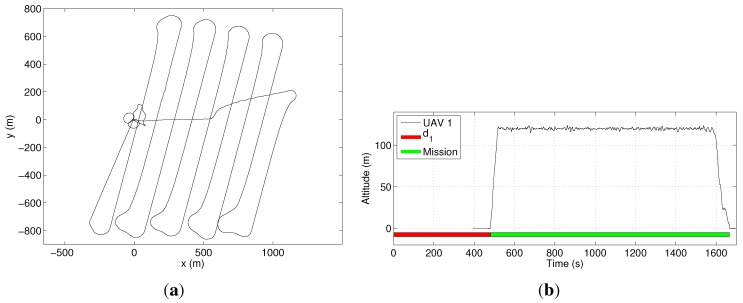
Coverage of the area in Figure 12 with a single UAV. (**a**) The UAV’s path projected on a cartographic reference system; (**b**) the UAV’s altitude. In this figure, $d_{1}$ is the UAV setup time.

In our first experiment, we use only one UAV (M=1) in order to establish a time reference to cover the area. Figure 13a shows the path followed by the UAV, while Figure 13b shows its altitude. In this second figure, it is possible to see the time when the UAV is effectively executing the mission and the setup time, when it is on the ground. The mission was completed in 27.7min, including 8min of setup time. In Figure 13a, a small overshoot can be noticed after each curve made by the UAV. This is due to the fact that the distance between the coverage rows is smaller than the minimum curvature radius of the UAV, which is 115m. The turn radius is a function of the maximum roll angle, which was limited to avoid the loss of the GPS signal on the curves, once the GPS is on one of the UAV wings.

**Figure 14:**
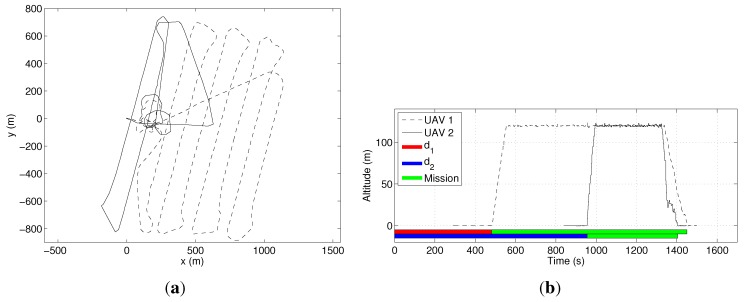
Coverage of the area in Figure 12 with two UAVs. (**a**) The UAVs’ paths projected on a cartographic reference system; (**b**) the UAVs’ altitude. In this figure, $d_{1}$ and $d_{2}$ are the cumulative setup times of UAVs 1 and 2, respectively.

In the second experiment, we used two UAVs (M=2). The optimizer distributed seven coverage rows for UAV 2 and only two rows for UAV 1. The mission time was reduced to 24.2min, including the setup time for each UAV. Notice that the mission time corresponds to the time spent by UAV 1 to cover the area, plus 8 min of setup time. The results for this mission are shown in Figure 14. A video illustrating this experiment can be seen at [23].

**Figure 15:**
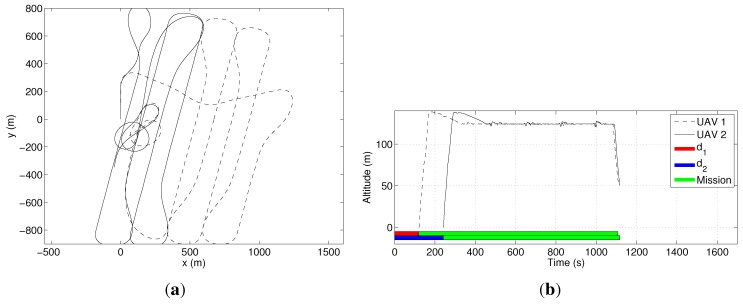
Coverage of the area in Figure 12 with two UAVs and a smaller setup time. (**a**) The UAVs’ paths projected on a cartographic reference system; (**b**) The UAVs’ altitude. In this figure, $d_{1}$ and $d_{2}$ are the cumulative setup times of UAVs 1 and 2, respectively.

For this specific area, it can be noticed that the gain in time to include an extra UAV was only 3min. Although one can think of a mission where 3min would make a difference, such as search and rescue operations, we can also see that in most situations, this reduction is not worth the cost of a second UAV. One way to reduce the coverage time even more is reducing the setup time by, for example, increasing the number of human operators working on a single UAV. We tested this strategy with a setup time of only two minutes. In this case, UAV 1 was assigned to five coverage rows, while UAV 2 was assigned to four rows. The mission time was reduced to 18.4min, which corresponded to a much larger economy of time if compared to the initial 27.7min. Data from this experiment are in Figure 15.

## 6. Conclusions and Future Work

This paper presented a methodology for the coverage and sensing of ground areas using fixed-wing UAVs. The main contribution of the method is that it explicitly and formally considers some practical problems that only appear during the deployment of the actual vehicles. The number of UAVs used in the task, for instance, is chosen as a function of the size and format of the area, the maximum flight time of the vehicles and, more importantly, the time needed to prepare and launch the UAV, which we call setup time. This time was never considered before in the solutions for this kind of problem, which frequently resulted in trivial solutions where the time of coverage is inversely proportional to the number of UAVs used. This is certainly not true in practice if the number of human operators is smaller than the number of UAVs to be launched.

It is important to mention that our methodology would not find a solution to the coverage problem in some situations, which include the ones where the number of UAVs and their battery life are small given the size of the area to be covered. In such a situation, it would be interesting to have a methodology that allows the UAVs to land, recharge and take-off again to complete the mission. This strategy is considered as future work.

It is also important to say that the UAV routing strategy proposed in this paper does not take into account possible collisions among the UAVs. To avoid collisions when the planned paths intersect, a velocity planner, such as the one proposed in [24], would be necessary. The addition of such a step to our architecture is left as a future development.
